# The Effect of Growth Hormone Administration on the Regulation of Mitochondrial Apoptosis *in-Vivo*

**DOI:** 10.3390/ijms160612753

**Published:** 2015-06-05

**Authors:** James Keane, Lotti Tajouri, Bon Gray

**Affiliations:** Faculty of Health Science and Medicine, Bond University, Gold Coast, Queensland 4227, Australia; E-Mails: ltajouri@bond.edu.au (L.T.); bgray@bond.edu.au (B.G.)

**Keywords:** growth hormone, mitochondrial apoptosis, Bcl-2 proteins, mito-miRNAs

## Abstract

The purpose of this study was to determine whether recombinant human growth hormone (rhGH) would show any significant effects on the expression of apoptosis regulating proteins in peripheral blood mononuclear cells (PBMCs). Additionally, the potential for post-transcriptional regulation of gene expression by miRNA was assessed in two cellular compartments, the cytosol and the mitochondria. Ten male subjects were subcutaneously injected with either rhGH (1 mg) or saline (0.9%) for seven consecutive days in a double-blinded fashion. Blood sampling was undertaken prior to treatment administration and over a period of three weeks following treatment cessation. Bcl-2 and Bak gene and protein expression levels were measured in PBMCs, while attention was also directed to the expression of miR-181a and miR-125b, known translational inhibitors of Bcl-2 and Bak respectively. Results showed that rhGH significantly decreased Bak protein concentrations compared to placebo samples for up to 8 days post treatment. While cytosolic miRNA expression was not found to be significantly affected by rhGH, measurement of the expression of miR-125b in mitochondrial fractions showed a significant down-regulation eight days post-rhGH administration. These findings suggest that rhGH induces short-term anti-apoptotic effects which may be partially mediated through a novel pathway that alters the concentration of mitochondrially-associated miRNAs.

## 1. Introduction

Apoptosis or “programmed cell death” is a physiologically favourable form of cell death which in addition to maintaining homeostatic control over cell populations, functions to remove damaged or diseased cells without incurring an inflammatory reaction [1,2]. Both “intrinsic” and “extrinsic” signalling pathways have been identified to mediate an apoptotic response [1]. The “intrinsic” pathway responds to sensors of intracellular damage resulting in the release of pro-apoptotic proteins from the mitochondrial inter-membrane space into cytosolic compartments [1]. The “extrinsic” pathway is characterized by extracellular ligand activation of plasma membrane located receptors and mediates intracellular caspase enzyme activation leading to apoptosis [1]. Mitochondrial mediated apoptotic signals are also initiated by extracellular stimulation which serves to propagate the caspase response, demonstrating the key and central roles of mitochondrial organelles in apoptosis [3]. Dysfunctional regulation of these apoptotic pathways is implicated in the development of pathological conditions such as cancer, diabetes, ischemia-reperfusion, autoimmune disorders, neurodegenerative diseases and acute organ failure [2,4,5,6].

Interestingly, *in vitro* recombinant human growth hormone (rhGH) administration has been well documented to induce anti-apoptotic effects following stimulation of both the intrinsically and extrinsically mediated apoptotic pathways. Anti-apoptotic effects were reported and demonstrated in various cell lines including peripheral blood lymphocytes, monocytes and pancreatic β cells [7,8,9]. In addition, insulin-like growth factor-1 (IGF-1), whose secretion from diverse tissues including the liver is regulated by growth hormone (GH) *in vivo* [10], shows well characterised survival effects both for doxorubicin challenged cardiomyocytes [11] and for neuroblastoma cells under hyperosmotic stress [12].

Despite these noted anti-apoptotic effects, supra-physiological *in vivo* concentrations of GH, arising from either disease states or rhGH administration in healthy individuals, have been shown to cause hyperglycemia, hypertriglyceridemia and in some cases to lead to the development of new onset diabetes [13]. Under such pathological conditions, cells are exposed to oxidative stress and damage which may predispose them to prematurely undergo apoptosis [14]. Sustained hyperglycemia has previously been reported to induce cardiomyocyte apoptosis in diabetes and animal models [15]. In light of the development of these pro-apoptotic conditions, even following the cessation of growth hormone abuse, the temporal extent of the anti-apoptotic effects of GH could have important implications for cellular survival.

In both extrinsic and intrinsic pathways, the release of cytochrome *c* (cyt *c*) from the inter-membrane space is widely regarded as the rate-limiting step towards initiation of the execution phase of apoptosis, the phase at which the cell is committed to programmed death [1]. While debate is ongoing as to whether cyt *c* release is instigated by permeabilization across the inner mitochondrial membrane (IMM) and the outer mitochondrial membrane (OMM) via the mitochondrial permeability transition pore (mtPTP) activation or simply by OMM permeabilization, there is a general consensus that the release of cyt *c* is regulated by the Bcl-2 family of proteins [2,16]. To date, 25 Bcl-2 family proteins have been identified, which exhibit either a pro-apoptotic or anti-apoptotic function [1]. It has been extensively demonstrated that the concentration ratio of the anti-apoptotic OMM based Bcl-2 proteins, Bcl-2 and Bcl-xL, and pro-apoptotic Bax and Bak proteins is an important determinant as to whether a cell undergoes apoptosis in response to either intrinsic or extrinsic stimuli [17,18].

Bcl-2 has been demonstrated to inhibit a wide array of apoptotic agents. Up-regulation of Bcl-2 gene expression in neural cells has been reported to result in decreased levels of intracellular reactive oxygen species (ROS) production, in addition to increased resistance to both mitochondrial dysfunction and cell death induced by oxidative stress [1]. The prevention of apoptosis upon death-receptor activation by GH administration in peripheral blood mononuclear cells (PBMCs) *in vitro* has been previously associated with increased Bcl-2 expression [7,9,19]. In contrast, over expression of Bak has been widely documented to accelerate the process of apoptosis in both murine and human cultured cell lines [20,21]. Homo-oligomerization of Bak is found to induce cyt *c* release from the mitochondrial inter-membrane space [16,22]. In contrast to cytosolic located Bax, Bak is perennially located within the OMM [16,22], explaining possibly why in single Bax^−/−^ knockout mouse embryonic fibroblasts (MEFs), Bak-dependent cells are found to exhibit significantly higher rates of apoptosis compared to Bax-dependent cells in Bak^−/−^ MEFs [16]. While synthesis of Bax is found to be significantly decreased in the presence of GH [23], surprisingly the effects of both GH and IGF-1 administration on the level of Bak gene and protein expression have, to date, not been investigated.

The respective down- and up-regulation of these pro- and anti-apoptotic proteins contributes significantly to the survival effects induced by GH and IGF-1. Interestingly, these two hormones have also been shown to induce changes in microRNA (miRNA) molecules in target cells and tissues involved in regulating protein expression [24]. Mature miRNAs are non-coding short single stranded RNA sequences of approximately 18–22 nucleotides in length, known to regulate gene expression post-transcriptionally [25]. Bcl-2 and Bak genes have been experimentally validated as targets for translational down-regulation by miRNAs miR-181a [26,27,28] and miR-125b [29,30] respectively. In addition to their apoptotic roles, miR-181a has been demonstrated to act as a potent inhibitor of cellular proliferation, while miR-125b has been shown to adversely affect cellular senescence and apoptosis [31,32]. Interestingly, whether or not GH or IGF-1 induces changes in the expression of these specific miRNAs has not been reported to date in the literature.

While miRNAs have predominantly been shown to exert their post-transcriptional influence in the cytosol, recently several studies have demonstrated the localisation of miRNA compartmentalised in mitochondria [33,34,35,36,37]. Interestingly, the level of expression of these mitochondrial-associated miRNAs (mito-miRNAs) has been shown to be independent of the total cellular miRNA expression profile [33,34]. Surprisingly, Kren *et al.* [34] have demonstrated that the mito-miRNA pool isolated from rat liver-derived mitochondria was not predicted to regulate any nuclear derived mRNA transcripts coding for mitochondrial located proteins. While the significance of these findings has yet to be elucidated, it has been speculated that mitochondria may serve as a miRNA reservoir [34]. Furthermore, the trafficking of these short nucleotide sequences between the mitochondrial compartment and the cytosol is thought to be under the regulation of cytosol located mRNA-processing bodies (P-bodies) [35]. It has been suggested that the storage and subsequent release of selective miRNA from mitochondria may act as a mechanism of intracellular signalling, exerting control over cellular processes such as apoptosis, proliferation and differentiation [33,34]. Indeed, the predicted gene-targets of some miRNA identified to be localized to mitochondria are consistent with those found to be down-regulated at the onset of cell death [34]. Furthermore, disruption of mitochondrial function has been reported to precede translational inhibition taking part in the apoptotic process [34]. Considering the known anti-apoptotic effects exhibited by rhGH administration, it is possible such anti-apoptotic roles might be mediated in part by mito-miRNAs.

The purpose of this study is to determine the effects on the regulation and expression of pro- and anti-apoptotic Bcl-2 family proteins in PBMCs following the cessation of a seven day programme of rhGH administration in healthy trained male subjects for a period of three weeks. Evaluation of Bcl-2 and Bak expression was determined for transcriptomic mRNA and translational protein levels from mitochondrial fractions. In addition, cytosolic expressions of miR-181a and miR-125b were determined to identify possible effects on their respective Bcl-2 and Bak target mRNA and protein expressions. As both miR-181a and miR-125b have previously been found to be significantly expressed in isolated mitochondria from human skeletal primary muscular cells [36], the present study therefore aimed at determining whether these two miRNAs were present in isolated mitochondria from the subjects PBMCs. Correlation analysis was performed to assess the possible relative contribution of rhGH induced expression of mito-miRNAs with Bcl-2 and BAK protein/mRNA levels. We hypothesized that the anti-apoptotic role of GH might be mediated through the activation of an intracellular signalling cascade implicating mito-miRNA recruitment and action.

## 2. Results

### 2.1. Bak/Bcl-2 Protein Concentrations

**Figure 1:**
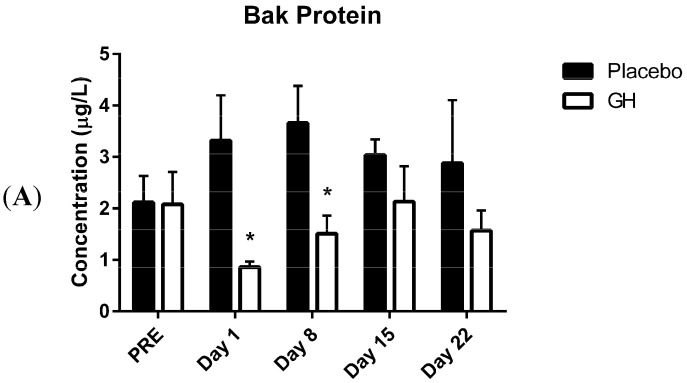

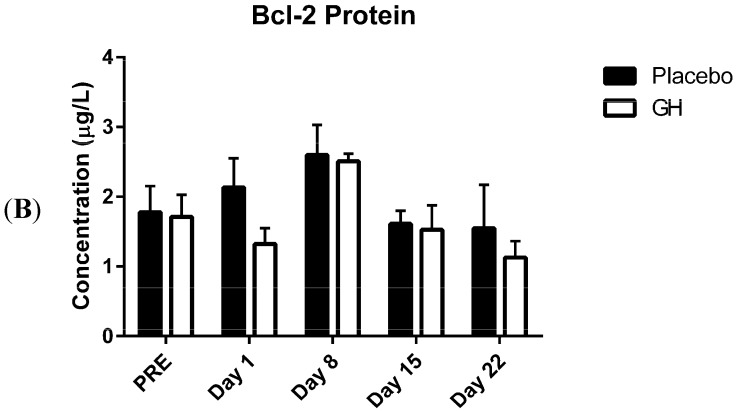
(**A**) Protein concentrations of Bak from peripheral blood mononuclear cell (PBMC) mitochondrial extracts in recombinant human growth hormone (rhGH) treated compared to placebo treated samples; (**B**) Protein concentrations of Bcl-2 from PBMC mitochondrial extracts in rhGH treated compared to placebo treated samples. (* *p* < 0.05 compared to placebo).

The pre-treatment measurements of Bak protein concentrations from mitochondrial fractions in rhGH and placebo groups were not significantly different from each other (Figure 1A). Interestingly, Bak protein concentrations were found to be significantly decreased in rhGH treated samples compared to placebo treated samples for measurements taken 1 day (mean difference ± SEM, 95% confidence intervals, *p*-values: −1.88 ± 0.75 μg/L, −3.66 to −0.99 μg/L, *p* ≤ 0.05) and eight days (−2.16 ± 0.91 μg/L, −4.32 to −0.09 μg/L, *p* ≤ 0.05) post-treatment. However, no significant differences were observed between treatment groups at 15 and 22 days post-treatment. No significant differences were observed in Bcl-2 protein concentrations from mitochondrial fractions between rhGH and placebo treated groups at any time point measured (24 h pre-treatment; 1, 8, 15 and 22 days post treatment) (Figure 1B). Cytosolic Bak and Bcl-2 protein concentrations were also analyzed but were found to fall below the detection limit of both assays and subsequently are not reported in this manuscript.

### 2.2. Bak/Bcl-2 mRNA Expression

**Figure 2:**
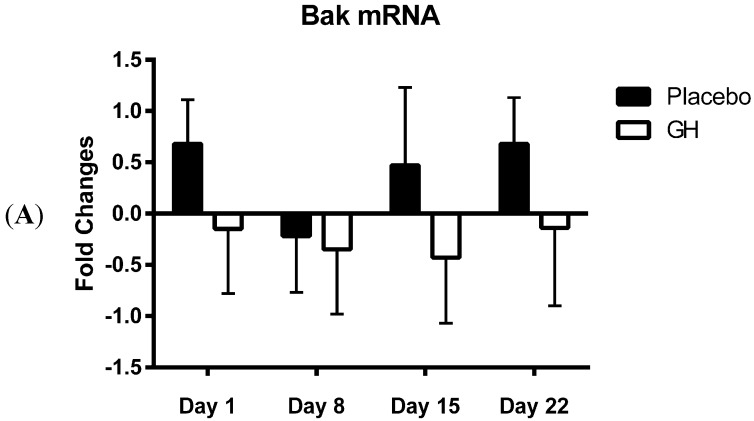

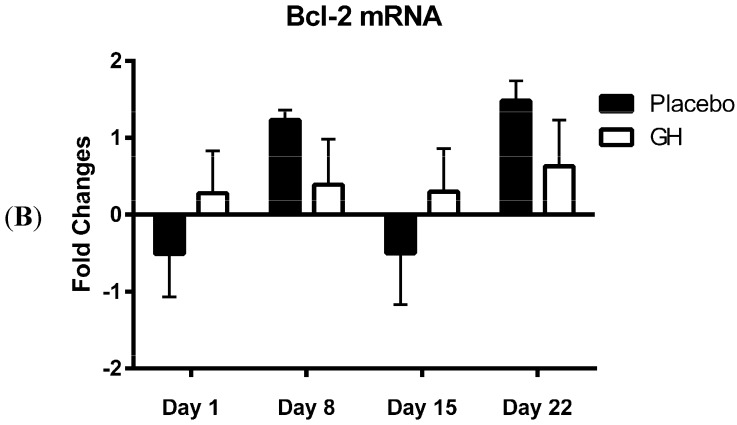
(**A**) Changes from baseline measurements in the expression of Bak mRNA levels from PBMCs in rhGH treated compared to placebo treated samples; (**B**) Changes from baseline measurements in the expression of Bcl-2 mRNA levels from PBMCs in rhGH treated compared to placebo treated samples.

Seven days of administration of rhGH (1 mg per day) was not found to exhibit any significant effect on Bak mRNA differences in expression from baseline levels for any measured time period (1, 8, 15 and 22 days following cessation of the treatment program) compared to placebo treated controls (Figure 2A). Similarly, rhGH treated samples were not found to have any significant effect on Bcl-2 mRNA changes in expression from baseline levels in comparison to placebo treated samples at any time point analysed post treatment (Figure 2B).

### 2.3. miR-125b/miR-181a miRNA Expression

**Figure 3:**
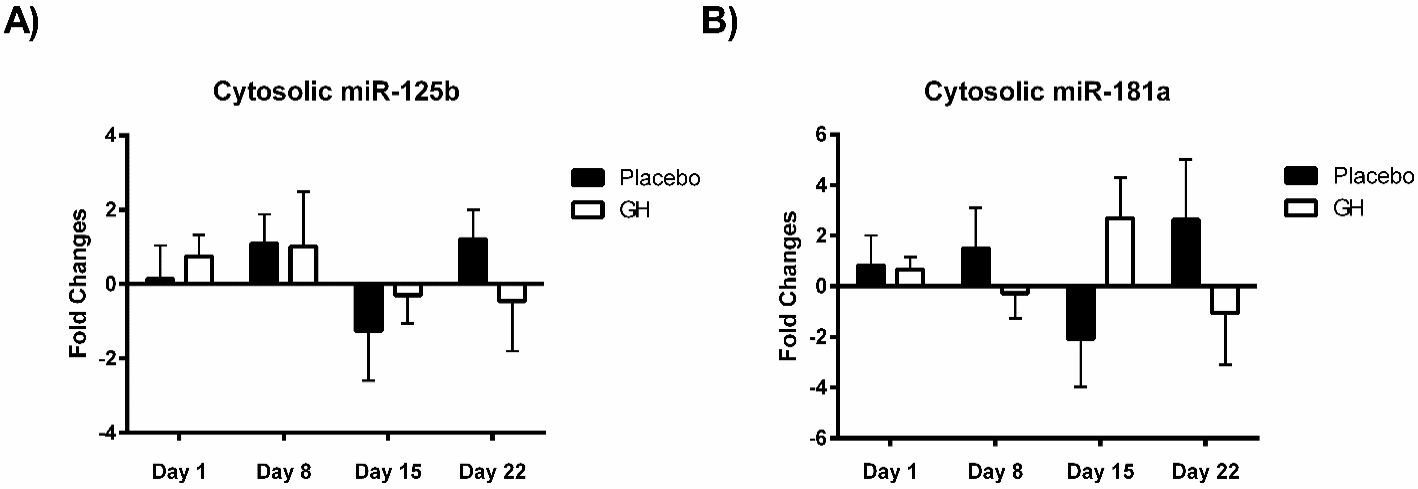

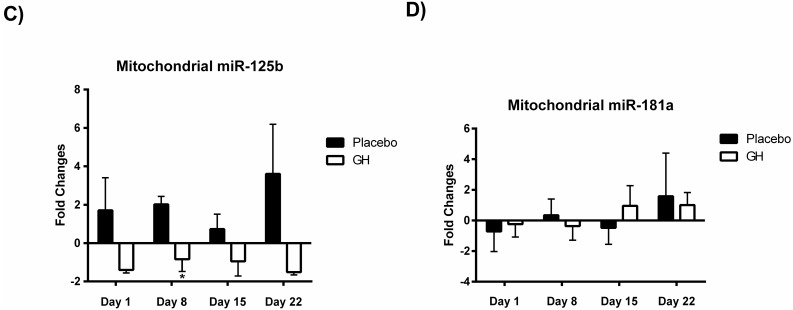
(**A**) Changes from baseline measurements in the expression of miR-125b miRNA levels from the cytosol of PBMCs in rhGH treated compared to placebo treated samples; (**B**) Changes from baseline measurements in the expression of miR-181a miRNA levels from the cytosol of PBMCs in rhGH treated compared to placebo treated samples; (**C**) Changes from baseline measurements in the expression of miR-125b miRNA levels from isolated PBMC mitochondria in rhGH treated compared to placebo treated samples; (**D**) Changes from baseline measurements in the expression of miR-181a miRNA levels from isolated PBMC mitochondria in rhGH treated compared to placebo treated samples. (* *p* < 0.05 compared to placebo).

No significant differences were observed in the level of expression of miR-125b from cellular lysate at any time point compared to baseline measurements between rhGH and placebo treated groups (Figure 3A). In addition, expression changes from baseline measurements for miR-181a from cellular lysate were not found to be significantly different between rhGH and placebo treated groups for any of the post-treatment time points (Figure 3B). The change in fold expression of miR-125b in mitochondrial fractions from baseline values was found to be significantly decreased in rhGH treated samples compared to placebo treated controls (−2.86 ± 0.74, −4.61 to −1.11, *p* ≤ 0.05) at 8 days post-treatment (Figure 3C). However no significant differences were observed between the treatment groups at 1, 15 or 22 days post-treatment. No significant differences were observed in the level of expression of miR-181a from mitochondrial fractions at any time point compared to baseline measurements between rhGH and placebo treated groups (Figure 3D).

No significant correlation was found for the expression differences from baseline levels between cytosolic miR-125b and either Bak mRNA or protein (Table S1). Similarly, cytosolic miR-181a fold expression changes did not significantly correlate with changes in the expression of either mRNA or protein Bcl-2 levels (Table S1). No significant correlation was found for the fold expression changes from baseline levels between mitochondrial associated miR-125b and either Bak mRNA or protein (Table S2). Additionally, mitochondrial associated miR-181a fold expression changes did not significantly correlate with changes in the expression of either mRNA or protein Bcl-2 levels (Table S2).

## 3. Discussion

Here we report, for the first time, significantly decreased protein concentrations of the pro-apoptotic Bcl-2 family member Bak in isolated PBMC mitochondria following rhGH administration. These decreases were observed at one and eight days post-treatment compared to placebo treated controls. While the level of expression of Bcl-2 plays an important role in determining whether or not a cell will undergo apoptosis, Zong *et al.* [38] demonstrated that the suppression of anti-apoptotic Bcl-2 family proteins was not sufficient to induce apoptosis in the absence of Bak and Bax. Indeed, considerable evidence exists to indicate that mitochondrial initiation of apoptosis is mediated via either a Bax or a Bak dependent pathway [38,39]. In addition to this, the Bak protein has been found to be a more potent inducer of cell death than Bax and it has been demonstrated that over expression of the Bak gene induces high levels of apoptosis regardless of the level of the anti-apoptotic Bcl-2 proteins (Bcl-2 and Bcl-xL) [16,21]. While it is well established that both GH and IGF-1 exert a significant down-regulation of the level of expression of the Bax gene [11,23], to our knowledge the effect of GH on Bak gene expression has not been demonstrated previously. In addition, conclusive evidence linking the effects of IGF-1 to Bak regulation has not been reported. Although Khan *et al.* [40] found that Bak protein expression was significantly down-regulated in mesenchymal stem cells (MSCs) following only one hour incubation in the presence of IGF-1 (50 ng/mL), this treatment was administered in combination with the survival factor, fibroblast growth factor-2 (FGF-2) (50 ng/mL). Thus, any effect witnessed on the regulation of Bak protein levels in that study could not solely be attributed to the actions of IGF-1. In addition, while Cui *et al.* [41] observed that *in vitro* administration of IGF-1 (100 ng/mL) significantly decreased the ratio of Bak to Bcl-xL mRNA expression in porcine parthenotes, they failed to report the quantitative level of expression of these genes. Therefore, considering that IGF-1 is a proven up-regulator of Bcl-xl expression [11,12], to what extent the hormone exerted an effect on Bak expression is unclear from their observations.

While rhGH was found in the present study to significantly decrease Bak protein levels at one and eight days post-treatment, no significant differences were observed in protein concentrations after either 15 or 22 days following the cessation of treatment. These results could have important implications in light of the considerable evidence for the association between chronic excesses in GH concentrations and apoptosis. Indeed, a recent publication from our laboratory [42] has demonstrated that while rhGH administration at physiological concentrations of up to 10 μg/L significantly decreases mitochondrial superoxide production, a well-known mediator of intracellular apoptotic signalling [43], this effect was not evident at higher concentrations. In addition to the observed metabolic alterations resulting from chronic elevations in GH and IGF-1 concentrations which can lead to the development pro-apoptotic conditions *in vivo* [13,14], case studies have reported that a prolonged history of rhGH abuse can lead to the development of cardiomyopathy and heart failure [44,45], conditions which are associated with deregulated apoptosis [46]. Significant increases in cardiomyocyte apoptosis has been reported in myocardial biopsies from patients with acromegaly, apparently contributing to cell loss and functional abnormalities in acromegalic cardiomyopathy [46,47]. Indeed, the degree of cardiac apoptosis was found to exhibit a significant positive relationship with both serum IGF-1 concentrations and the reported duration of acromegalic disease [47]. In light of these observations, the finding that the anti-apoptotic effects exerted by rhGH on Bak protein levels does not persist, at least up until 15 days following the cessation of treatment could have significant health consequences for individuals who are administering rhGH at supra-physiological concentrations.

Bak mRNA was observed to be down-regulated following rhGH administration at all time-points compared to placebo treated controls. However unlike the decrease in Bak protein concentrations these results were not found to be significant. Our results at the transcriptomic and proteomic level for Bak therefore show a “mismatch” of expression. However, it has been experimentally validated that multiple miRNAs can target the same mRNA target, suggesting it is likely that there are a number of miRNAs expressed within a cell at any given time that will determine the overall change in the expression and function of specific genes [28,48]. For instance, in addition to miR-125b, Bak mRNA is also known to be directly targeted by miR-125a, miR-26a and miR-29b [49,50,51]. MiR-125b has been shown to significantly decrease Bak mRNA inducing mRNA degradation [30], however not all miRNAs function in this manner. Indeed, certain miRNAs simply act to repress gene translation, leaving the mRNA molecule intact [25,29]. Several studies have observed protein silencing by miRNA with or without a change of mRNA levels [52,53,54]. Thus, it is possible that the dual effect of mRNA degradation and translational inhibition, resulting from a combinational targeting of multiple miRNAs on Bak mRNA, may be responsible for the significant changes observed in Bak protein concentrations following rhGH administration.

Considering the effect of rhGH on Bak protein concentrations, it is surprising that the level of expression of miR-125b in cellular lysate was not found to be significantly affected by rhGH treatment compared to placebo treated controls. In addition, miR-125b expression levels were not correlated with either Bak mRNA or protein expressions in both treatment groups. These results would suggest that GH does not exert its effects on the level of expression of Bak through the regulation of miR-125b, possibly indicating that other miRNAs known to target Bak mRNA may play a role [49,50,51]. Indeed it should be noted that several other sources of modulation could ultimately be responsible for the changes observed in Bak protein concentrations. These modulations can occur at different levels of the regulation of gene expression and may include changes in transcriptional initiation by means of transcription factor and co-regulator proteins [55]; post-transcriptional modulation such as regulation of mRNA decay by the means of RNA-binding proteins [56]; and translational regulation controls by mechanisms implicating the eukaryotic translation initiation factor activation upon phosphorylation [57].

However, evidence strongly suggests that the control of mRNA expression by miRNA is combinatorial, meaning that it is not a one miRNA to one mRNA interaction but rather a combination of multiple miRNAs targeting the same mRNA that determines the level of translational regulation [28,48]. The combinational effect of multiple miRNAs targeting a specific mRNA could have a considerably higher biological significance than an individual miRNA targeting the same mRNA [48]. Thus, the expression of miR-125b may not, on its own, be considered sufficient to induce the observed decreases in Bak protein concentrations. However, miR-125b acting in combination with other miRNAs may contribute to a significant down-regulation of Bak protein expression.

An alternative explanation is that rhGH induced significant increases in miR-125b levels early in the treatment program and that these elevations in expression had subsided at the post-treatment time points analysed. It has been reported that the concentration of an individual miRNA relative to its mRNA targets is likely to be more important to mediating its biological effects than its absolute copy number [27]. In addition, although miRNAs are generally assumed to have a very long half-life, corresponding to many hours or even days, such a slow turnover may not be a universal feature of miRNAs since they often play a role in rapid developmental transitions which require a more active miRNA metabolism [58]. While miR-125b was reported to have a half-life of 225 h (~9 days) in mouse embryonic fibroblasts, the same miRNA was reported to exhibit rapid turnover (4–6 h) following lipopolysaccharide (LPS) stimulation in murine phagocytes and human dendritic cell lines [59,60,61]. Thus, it is plausible that rhGH induced significant increases in the expression of miR-125b early in the treatment program which, as a result of increased turnover, were subsequently down-regulated relative to decreases in Bak protein concentrations.

In the present study, expression levels of Bcl-2 mRNA and protein in PBMCs were not found to be significantly affected by rhGH administration. While this finding was unexpected, Bcl-2 protein concentrations have previously been found to remain unaffected in animal based trials following GH administration *in vivo* [23,62]. Cuesta *et al.* [23] examined the effects of four weeks rhGH treatment, administered subcutaneously at a dosage of 2 mg/kg/day, on the regulation of Bcl-2 family proteins in senescence-accelerated mice (SAM), a murine model of accelerated aging. While they reported that the ratio between anti-apoptotic (Bcl-2) and pro-apoptotic (Bax and Bad) proteins was significantly enhanced following GH administration, this was attributed to a decrease in the expression of pro-apoptotic protein levels as the expression of Bcl-2 was found to be unaffected. Liang *et al.* [62] investigated, *in vivo,* the effect of six days rhGH treatment on a human gastric cancer cell line (BGC823 cells) following their inoculation to induce tumor xenografts in nude mice. They reported that the level of Bcl-2 protein expression in xenograft tumors was not significantly different between mice treated with rhGH (2 International Units (IU) per kg per day) and saline administered controls.

MiR-181a is one of several miRNAs that directly target and repress translation of Bcl-2 [26,28]. Considering no rhGH mediated effect on the Bcl-2 level of expression was observed in the current study, it is not surprising that no significant difference was observed in miR-181a expression in cellular lysate in both rhGH and placebo treated subjects. In addition, miR-181a expression levels were not correlated with either Bcl-2 mRNA or protein expressions in both treatment groups.

The presence of four miRNAs have been identified in isolated mitochondria in the present study, adding PBMCs to the short list of cell types that have been found to contain mito-miRNA [33,34,36,37]. To date, miRNAs have been specifically associated with mitochondria isolated from rat liver [34], mouse liver [33], human HeLa epithelial cells [37] and human skeletal muscle cells [36]. In addition to miR-181a and miR-125b, the two miRNAs tested as endogenous references, miR-24 and miR-92 were found to be mitochondrially located. While miR-181a, miR-125b and miR-24 have all previously been documented in isolated mitochondria from human skeletal muscular cells [36], to our knowledge this is the first time that miR-92 has been shown to be localised to in mitochondria.

No significant correlations were found to exist between miR-181a or miR-125b expression levels in mitochondrial fractions and their respective mRNA targets in either treatment group. As miR-181a and miR-125b have been experimentally validated to target Bcl-2 [26,27,28] and Bak [29,30] respectively, that no association was detected between their expression profiles is likely a reflection of the small sample size utilized in the present study. Expression levels of miR-181a from mitochondrial fractions were not found to be significantly affected in rhGH treated subjects compared to placebo treated controls. However miR-125b was observed to be down-regulated in isolated mitochondria following rhGH administration at all time-points measured. Furthermore, this down-regulation was found to be significant at eight days post-treatment in comparison with the placebo group. Thus, the results from this study suggest that rhGH may exert a regulatory effect on the expression levels of at least some mito-miRNAs. However, identifying what the significance of this rhGH induced effect may be and how its regulation is mediated remain important issues which must be addressed.

It has been hypothesized that post-transcriptional regulation via mito-miRNAs might provide a sensitive and rapid mechanism to fine tune the level of protein expression from the mitochondrial genome in response to changes in cellular metabolic demands [37]. Indeed, miRNAs identified in isolated mitochondria from HeLa cells have been predicted *in-silico* to target mitochondrial RNA [37]. In addition, argonaute 2 (AGO2), the principal active protein of the cytoplasmic miRNA incorporated RNA-induced silencing complex (miRISC), has been found in several studies [33,36,37] to be present in isolated mitochondrial fractions. GH mediated signalling pathways have been demonstrated to directly target mitochondria, exerting regulatory control over the activity of electron transport chain (ETC) complexes and the rate of ATP synthesis [63,64]. Thus, it is possible that rhGH induces changes in mitochondrial function through mito-miRNA regulated changes in the expression of mitochondrial proteins.

Another hypothesis is that mitochondria serve as a storage site for miRNAs [34,35,65]. Both Kren *et al.* [34] and Barrey *et al.* [36] identified mito-miRNAs which exhibited no obvious mitochondrial mRNA targets, nor were they found to be complementary to nuclear mRNAs encoding mitochondrial proteins. In addition, all mito-miRNAs identified to date have predicted cytoplasmic protein targets with many of these found to be involved in the regulation of cellular processes such as apoptosis, proliferation and differentiation [33,34,65]. Thus, it is conceivable that a select group of miRNAs are sequestered into mitochondria for storage and that their signal mediated release into the cytoplasm mediates an additional level of control over cellular processes [34].

It has been proposed that mito-miRNAs released into the cytoplasm are trafficked into P-bodies, cytoplasmic granules involved in mRNA degradation that also contain the necessary machinery for translational inhibition [65,66]. In fact, it has been reported that P-bodies establish frequent and prolonged contacts with mitochondria [65,66]. Huang *et al.* [66] observed that 50%–70% of P-bodies were found to associate with mitochondria in established human cell lines, indicating that their interaction is not the result of a random association. In addition, the association between mitochondria and P-bodies is reported to be dynamic, with fluorescence microscopy imaging showing that more than 80% of P-bodies establish a link with mitochondria at least once within a three minute interval, with a median duration of eighteen seconds [65,66]. Whether this transient association involves the binding of P-body proteins to proteins on the surface of the OMM remains to be determined, however the proximity between the two organelles does make possible the exchange of metabolites, RNAs and proteins [65]. Furthermore, experimental evidence has arisen to suggest that cytosolic ribosomes bound to mitochondria may control a co-translational import process for at least some nuclear encoded mitochondrial transcripts and it is plausible that mitochondrial P-body interactions allows for regulation of these membrane bound mRNAs [66,67,68]. In the present study, the pre-treatment of isolated mitochondria with RNase indicates that all identified mito-miRNAs are located within the organelle as those associated with membrane bound P-bodies and ribosomes would have been destroyed. Thus, in order to determine whether mito-miRNAs are released from mitochondrial storage and subsequently associate with membrane bound granules, further studies incorporating the analysis of both RNase treated and untreated mitochondrial fractions are warranted.

In summary, the present study observed significant decreases in Bak protein concentrations following rhGH administration which could not be solely attributed to changes in the expression of miR-125b. However, that difference in Bak levels did not persist past eight days post-treatment. In light of the noted association between chronic excesses in GH concentrations and apoptosis, these findings could have implications that are detrimental to the health of any individual taking rhGH at supra-physiological concentrations for performance enhancing purposes. In addition, our results suggest that rhGH may exert an effect on the expression of at least one of the mito-miRNAs investigated, although how this effect is mediated remains to be determined. The data presented in this pilot study points towards the existence of GH induced anti-apoptotic effects which may in part be mediated via changes in the expression of mito-miRNAs. These preliminary and interesting observations warrant further investigation.

## 4. Experimental Section

### 4.1. Subjects

Ten healthy resistance trained male subjects (mean ± SEM: age = 25.40 ± 1.22 years, height = 1.80 ± 0.16 m, body mass = 78.92 ± 1.52 kg, BMI = 24.32 ± 0.53 kg/m^2^) were recruited to participate in the study which was approved by the Bond University Human Research Ethics Committee (BUHREC-RO597 (7 April 2011)). All subjects had both the nature of the study and the associated risks involved explained to them prior to providing written informed consent. Resistance trained was defined as the undertaking of a resistance exercise program, 3–5 days per week for a period of at least 12 months. Exclusion criteria included smoking, the use of therapeutic, recreational or performance enhancing drugs, including anabolic steroids and rhGH, up to 12 months prior to participation in the study, the use of prescription medication, the presence of diabetes, cardiovascular disease, acromegaly or any diagnosed condition which would contra-indicate either participation in resistance exercise or the administration of rhGH.

### 4.2. Reagents Used

Magnetic-activated cell sorting (MACS) Mitochondrial Isolation kits were purchased from Miltenyi Biotec (Auburn, CA, USA). RNase-free DNase I and Total RNA/Protein purification kits were purchased from Norgen Biotek (Thorold, ON, Canada). Miscript II RT kits, SYBR Green PCR kits and Hs-RNU6-2, Hs-MiR-24, Hs-MiR-92, Hs-MiR-125b and Hs-MiR-181a primer assays were purchased from Qiagen (Donchaster, Australia). Custom primers for Bcl-2, Bak, Glyceraldehyde 3-phosphate dehydrogenase (GAPDH), Hyperparathyroidism 1 (HRPT1) and 18s, as shown in Table 1, were synthesised by Geneworks (Thebarton, Australia). iQ SYBR Green supermix was purchased from Biorad (Hercules, CA, USA). Ribonuclease A and protease inhibitor cocktail were purchased from Sigma Aldrich (St. Louis, MO, USA), while RNasin PLUS RNase inhibitor was purchased from Promega (Madison, WI, USA). Human Bcl-2 platinum ELISA kits and human Bak ELISA kits were purchased from eBiosciences (San Diego, CA, USA) and USCN Life Science Inc. (Wuhan, China) respectively. Pierce bicinchoninic acid (BCA) protein assay kit was purchased from Thermo Scientific (Rockford, IL, USA). RNase/DNase free water and phosphate buffered saline (PBS) (pH 7.4) were purchased from Invitrogen (Carlsbad, CA, USA). Ficoll Paque PLUS was obtained from GE Healthcare (Rydalmere, Australia). Finally, Genotropin was purchased from Pfizer (Sydney, Australia).

**Table 1:** Primer sequences used for each mRNA gene product and the corresponding size of the PCR amplicon generated.

Gene	Unigene ID	Forward Sequence (5′–3′)	Reverse Sequence (5′–3′)	Amplicon Length (bp)
*18S*	100008588	TTCGAGGCCCTGTAATTGGA	GCAGCAACTTTAATATACGCTATTGG	123
*GAPDH*	2597	CTCTGCTCCTCCTGTTCGAC	ACCAAATCCGTTGACTCCGAC	108
*HRPT1*	3278	GCTGAGGATTTGGAAAGGGTG	CAGAGGGCTACAATGTGATGG	112
*Bcl-2*	596	CATCCAGTACCTTAAGCCCTG	CTCAGACAGAGCCAGTATTGG	83
*Bak*	578	GAGATGGTCACCTTACCTCTG	GCAACATGGTCTGGAACTCTG	117

### 4.3. Experimental Design

Subjects were randomly assigned to either a treatment group (rhGH; *n* = 5 years) who received 1 mg (mean ± SEM: 12.76 ± 0.49 μg/kg/day) of Genotropin, a recombinant human growth hormone which exhibits a complete sequence homology to the 22 kDa hGH isoform, or a placebo group (P; *n* = 5 years) who were administered with a saline solution (0.9% sodium chloride) for a period of seven consecutive days. Daily injections were administered subcutaneously in a double blind manner. Prior to each injection all participants received a standardized meal (protein shake) followed by a 15 min rest period. Blood samples were collected from each subject 24 h prior to treatment administration as well as 1, 8, 15 and 22 days post-treatment.

### 4.4. Blood Sample Collection and Peripheral Blood Mononuclear Cell (PBMC) Isolation

Subjects arrived for sample collection in a post-prandial state. Blood samples were taken from subjects resting in a supine position, prior to insertion of a catheter into the antecubital vein with 20 mL of blood being drawn into 10 mL EDTA Vacutainer blood collection tubes (Becton Dickinson Biosciences, San Jose, CA, USA). Collected EDTA blood was diluted in an equal volume of PBS (pH = 7.4) and layered over Ficoll-Paque PLUS at a ratio of 2:1. Samples were subsequently centrifuged at 450× *g* for 30 min to achieve separation of PBMCs from whole blood.

### 4.5. Mitochondrial Isolation and Cytosolic RNA Decontamination

Mitochondria were isolated from PBMCs using a magnetic antibody cell sorting method (MACS). Briefly, PBMCs were resuspended in 100 µL cell lysis buffer in the presence of 1 µL of protease inhibitor and homogenised 10 times by passage through a 27 gauge needle. The mitochondria in the cell lysate were subsequently magnetically labelled with microbead conjugated anti-TOM22 antibodies. The antibody labelled cell lysate was then loaded into a column placed in a magnetic field separator. Flow-through cell lysate was kept for later RNA extraction and magnetically labelled mitochondria bound to the column were washed as per manufacturer’s instructions. Mitochondria were finally eluted following the removal of the column from the magnetic field. Isolated mitochondria were further pelleted by centrifugation at 13,000× *g* for 2 min at 4 °C and resuspended in 200 µL RNase A solution (Ribonuclease A concentration: 10 µg/mL) at 37 °C for one hour to remove any possible residual cytosolic RNA molecules residing outside intact mitochondria. Post RNase incubation, mitochondria were pelleted a second time by centrifugation at 13,000× *g* for 2 min at 4 °C and resuspended in 100 µL mitochondrial storage buffer. RNase activity was stopped by the addition of 5 µL RNasin PLUS.

### 4.6. Total RNA and Protein Extraction from Cytosolic and Mitochondrial Lysates

Stored isolated mitochondria were pelleted by centrifugation at 13,000× *g* for 2 min at 4 °C, resuspended in 350 µL lysis solution containing high concentrations of chaotropic denaturant, for rapid inactivation of RNases and proteases, and vortexed vigorously to produce a mitochondrial lysate. Meanwhile the collected cell lysate was treated with 2 µL RNasin Plus and 1 µL protease inhibitor cocktail to protect the cytosolic RNA fraction from degradation. Both cytosolic and mitochondrial lysates were subsequently mixed with isopropanol at a ratio of 2.33:1. Total RNA purification of cytosolic and mitochondrial lysates was performed by spin column chromatography using a proprietary silicon carbide (SiC) resin (Norgen Biotek Corporation, Thorold, ON, Canada). The flowthrough from mitochondrial lysate was retained and stored at −80 °C for later analysis of mitochondrial proteins. Samples were subsequently treated with RNase free-DNase I for 15 min for the removal of all traces of residual DNA from columns prior to column washing and elution of purified RNA as per manufacturer’s instructions. Finally, total cytosolic and mitochondrial RNA was quantified on a spectrophotometer (Nanodrop 1000, Thermo Scientific).

### 4.7. cDNA Synthesis from Cytosolic and Mitochondrial RNA

Reverse transcription of total RNA into cDNA was performed using the miScript II RT kit from Qiagen. Mitochondrial and cytosolic RNA were transcribed separately with 12 µL of purified RNA being used in each 20 µL reaction volume. The miScript HiFlex Buffer was used for cDNA synthesis which was used as a template for real-time PCR (RT-PCR) gene expression analysis and quantitation of both miRNA and mRNA. Mature miRNAs were polyadenylated by poly (A) polymerase and reverse transcribed into cDNA using modified common oligo-dT primers as these primer sequences hold a 3′ degenerate “anchor” and a universal tag sequence on the 5′ end which allowed for amplification of mature miRNA by RT-PCR. All non-miRNA RNA types were converted into cDNA using usual oligo-dT and random primers. These samples were reverse transcribed at 37 °C for 60 min prior to being heated at 95 °C for 5 min for inactivation of miScript reverse transcriptase enzyme.

### 4.8. Real-Time Polymerase Chain Reaction (RT-PCR) Analysis

RT-PCR assays were performed using a Corbett Rotor-gene 6000 (Qiagen). For relative quantification of mRNA gene expression, lysate-derived cDNA was mixed with iQ SYBR Green supermix (Biorad) and various sets of gene specific primers in a 25 µL reaction volume. The forward and reverse sequences of the primers used for each gene product are listed in Table 1. After confirming comparative PCR efficiency with experimental primers, GAPDH was used as an endogenous reference gene for normalisation of target mRNA expression. RT-PCR reactions were conducted at 94 °C for 12 min, followed by 40 cycles at 94 °C for 30 s, 59 °C for 30 s and 72 °C for 30 s.

For relative quantification of miRNA expression, both lysate and mitochondrially derived cDNA were mixed with Quantitect SYBR Green PCR master mix and miScript universal reverse primer (Qiagen) in combination with target-specific miScript forward primers in a 20 µL reaction volume as per manufacturer’s instructions. The predesigned forward primers, purchased from Qiagen, were as follows: Hs-RNU6-2 (Cat No: MS00033740), Hs-MiR-24 (Cat No: MS00006552), Hs-MiR-92 (Cat No: MS00006594), Hs-MiR-125b (Cat No: MS00006629) and Hs-MiR-181a (MS00008827). Amplicon lengths of 85–87 bp were expected for all mature miRNA PCR products. RNU6-2 served as an endogenous reference miRNA for normalisation of target lysate-derived miRNA expression. Concentrations of mitochondrial derived cDNA from five samples were normalised against sample mitochondrial protein concentrations. These samples were subsequently analysed by RT-PCR as described for determination of the most appropriate endogenous control miRNA for normalisation of target mitochondrial miRNA expression. Among three possible candidates (RNU6-2 [69], Hs-MiR-24 [70] and Hs-MiR-92 [71]), MiR-92 was found to be the most stably expressed. RT-PCR reactions were conducted at 95 °C for 15 min, followed by 40 cycles of 94 °C for 15 s, 55 °C for 30 s and 70 °C for 30 s. The cycle threshold values obtained from all RT-PCR experiments are presented as supplementary material (Table S3). The relative expression of mRNA and miRNA were calculated using the comparative *C*_t_ (ΔΔ*C*_t_) method. The results are presented as log_2_ fold differences of each target mRNA and miRNA in all post-treatment samples relative to baseline samples. All reactions were performed in triplicate and no-template control reactions were run simultaneously with each PCR assay.

### 4.9. Determination of Bcl-2 and Bak Protein Concentrations

Quantification of total mitochondrial protein concentration was determined using the bicinchoninic acid (BCA) assay. Samples were incubated at 37 °C for 30 min in an alkaline medium in the presence of BCA and cupric sulphate. The reduction of Cu^2+^ ions from cupric sulphate to Cu^+^ occurs in proportion to the amount of protein present in the sample. Two molecules of BCA chelate each Cu^+^ ion formed. The product exhibits a high absorbance at 562 nm, which was read on a Modulus microplate reader (Turner Biosystems, Sunnyvale, CA, USA). A standard curve, developed from known concentrations of BSA, was used to determine sample protein concentrations. Total protein concentration was subsequently normalised for all samples prior to analysis of Bcl-2 and Bak levels.

Bcl-2 and Bak concentrations from mitochondrial protein samples were assessed by enzyme-linked immunosorbent assay (ELISA). Briefly, samples were incubated on antibody coated microtitre plates together with biotin-conjugated antibodies, both of which were specific to the analyte of interest. Subsequently, streptavidin-conjugated horseradish peroxidase (HRP) was added which binds to biotin-conjugated antibodies. Removal of unbound antibody and enzyme by microplate washing allows for the development of a colour change in proportion to the concentration of analyte present in sample wells, following the addition of 3,3ʹ,5,5ʹ-tetramethylbenzidine (TMB), a substrate of HRP. The enzyme-substrate reaction was terminated by addition of sulphuric acid and absorbance was measured at 450 nm on a Modulus microplate reader (Turner Biosystems). The optical density of analysed samples was used in conjunction with the development of standard curves for the calculation of Bcl-2 and Bak concentrations, according to manufacturers’ instructions. All samples were analysed in duplicate. Respective intra- and inter-assay coefficients of variability were 5.36% and 7.94% for Bcl-2 and 6.49% and 8.45% for Bak.

### 4.10. Statistical Analysis

All data is reported as mean ± SEM. A two-way ANOVA with repeated measures was used to determine whether significant differences exist between the two treatment groups (rhGH and placebo) for fold changes in mRNA and miRNA expression and mitochondrial protein concentrations. Bonferroni’s multiple comparisons were used for *post hoc* analysis to identify the location of significant differences between conditions. Pearson’s product moment correlational analysis was used to determine the existence of any significant relationships between the analysed variables (SPSS Inc., PAWS Statistics Version 18, Chicago, IL USA). Statistical significance was accepted at the *p* < 0.05 level of confidence.

## Supplementary Materials

Supplementary materials can be found at http://www.mdpi.com/1422-0067/16/06/12753/s1.
