# Supplementary material for: The Effect of Growth Hormone Administration on the Regulation of Mitochondrial Apoptosis in-Vivo
Source: Int J Mol Sci. 2015 Jun 5;16(6):12753–72. doi: 10.3390/ijms160612753 (PMC4490471; doi:10.3390/ijms160612753)
Supplement: Supplementary file 1 [file ijms-16-12753-s001.zip › ijms-81968-Supplementary Information/ijms-81968-Tables S1 and S2.pdf]

Supplementary Information

Table S1. Pearson's correlational co-efficients of cytosolic associated miRNA fold changes in expression against mRNA/Protein fold changes in expression for the corresponding gene of interest in placebo and rhGH treated groups.

**Cytosolic miRNA**		**Placebo**	**GH**
miR-181a	Bcl-2 mRNA	0.411 (p = 0.080)	0.402 (p = 0.088)
	Bcl-2 Protein	−0.108 (p = 0.650)	−0.217 (p = 0.372)
miR-125b	Bak mRNA	0.345 (p = 0.137)	−0.115 (p = 0.638)
	Bak Protein	0.162 (p = 0.495)	0.052 (p = 0.834)

Table S2. Pearson's correlational co-efficients of mitochondrial associated miRNA fold changes in expression against mRNA/Protein fold changes in expression for the corresponding gene of interest in placebo and rhGH treated groups.

**Mitochondrial miRNA**		**Placebo**	**GH**
miR-181a	Bcl-2 mRNA	−0.198 (p = 0.480)	0.369 (p = 0.131)
	Bcl-2 Protein	−0.155 (p = 0.567)	−0.313 (p = 0.207)
miR-125b	Bak mRNA	−0.079 (p = 0.741)	0.374 (p = 0.127)
	Bak Protein	−0.220 (p = 0.351)	−0.167 (p = 0.508)
